# Immune-related inflammatory gene in hypertrophic scar: prognostic and molecular mechanisms via integrated machine learning-WGCNA analysis

**DOI:** 10.3389/fimmu.2025.1645721

**Published:** 2025-10-28

**Authors:** Yufang Liu, Huiling Li, Chunfeng Zhao, Wang Xin

**Affiliations:** ^1^ Department of Burn and Wound Repair, Weifang People’s Hospital, Weifang, Shandong, China; ^2^ Department of Pediatric Surgery, Weifang People’s Hospital, Weifang, Shandong, China

**Keywords:** hypertrophic scar, immune-related inflammatory gene, signature genes and mode, immune cell infiltration, machine learning, validation analysis

## Abstract

**Introduction:**

This research aimed to explore key immune-related inflammatory genes and associated molecular mechanisms on hypertrophic scar (HTS), to provide new perspectives for disease prognosis and diagnosis.

**Methods:**

The gene expression profiles were obtained from the public GEO database. The immune-related inflammatory genes were identified based on DEGs from HTS vs. normal samples, immune-related genes explored by WGCNA, as well as inflammation-related genes from the database. Signature genes were screened using machine learning methods, followed by nomogram validation. Then, the immune infiltration, GSEA pathway analysis, target drug prediction and interaction analysis associated with signature genes were further investigated. Finally, validation analysis was performed using tissue samples from HTS patients to verify the expression of signature genes.

**Results:**

A total of 73 differentially expressed immune-related inflammatory genes were identified. Through three machine learning analysis approaches, four signature genes (COL1A1, A2M, TIMP1, and COL1A2) were identified, and they exhibited strong prognostic value in nomogram analysis. Immune infiltration and GSEA analysis revealed significant associations between these signature genes and Nature killer T cells, as well as the ECM receptor interaction pathway. Validation analysis via qRT-PCR and Western blot confirmed significant differential expression of all signature genes in HTS compared with normal skin tissues. Furthermore, transfection of HTS fibroblasts with si-COL1A1 not only reduced COL1A1 expression but also suppressed fibroblasts proliferation while promoting apoptosis, indicating that COL1A1 promotes proliferation and inhibits apoptosis in HTS fibroblasts.

**Discussion:**

The immune-inflammation related genes COL1A1, A2M, TIMP1, and COL1A2 were identified as novel signature genes in HTS. The nomogram established based on these genes demonstrated high clinical diagnosis value. These findings provide evidence for early diagnosis and personalized therapeutic strategies in HTS management.

## 1 Introduction

Hypertrophic scar (HTS) is a prevalent fibrotic skin disorder that typically arises following trauma, surgery, or burns, characterized by excessive proliferation of cutaneous tissue and abnormal collagen deposition (1). Although HTS does not pose a direct life-threatening risk, it can cause functional impairments and significant psychological distress (2). Currently, it is more efficient to prevent HTS than treat them, and reliable biomarkers for early identification and prediction of HTS can considerably impact the overall outcome.

The pathogenesis of HTS is complex, involving multiple cell types and molecular pathways, particularly dysregulation of immune-inflammatory responses (3). For example, the accumulation of pro-fibrotic immune cells, including M2 macrophages, dendritic cells, mast cells, and Th2 cells, induces the transition of fibroblasts to myofibroblasts through the transforming growth factor-beta1 (TGF-β1) signaling pathway (4). Furthermore, inflammation is also one of the determining factors for wound healing, and the intensity of inflammation is positively correlated to final scar sizes (5–7). Therefore, immune-related inflammatory responses play a pivotal role in the formation and progression of HTS (8). Studies have demonstrated significant immune cell infiltration and up-regulation of inflammatory factors in HS tissues, suggesting that dysregulation of the immune microenvironment may be a core driver of HTS pathogenesis (9). For instance, abnormal accumulation of immune cells (e.g., T cells and B cells) in HTS tissues has been widely reported (10). These cells promote fibroblast activation and excessive collagen deposition through releasing pro-inflammatory factors and growth factors. Additionally, aberrant activation of inflammation-related signaling pathways such as NF-κB is recognized as a crucial mechanism in HTS formation (11). At present, the role of immune-related inflammatory biomarkers has attracted attention, but most exhibit limitations including small sample sizes and methodological constraints (12, 13). In recent years, the application of machine learning algorithms in biomedicine has expanded significantly, showing strong potential for biomarker screening and validation (14). By integrating multi-omics data via machine learning models, researchers can identify disease-associated key genes from extensive gene expression datasets and develop predictive models. However, machine learning applications in HTS research remain nascent, with few studies targeting immune-related inflammatory biomarkers screening and validation.

In this study, we aim to systematically identify and validate immune-related inflammatory biomarkers in HTS using multiple machine learning algorithms, integrated with bioinformatics analysis and experimental validation. Furthermore, the biological functions of these biomarkers will be verified through *in vitro* studies, with the ultimate goal of providing novel insights and potential therapeutic targets for HTS management.

## 2 Materials and methods

### 2.1 Microarray data and data preprocessing

Profiles associated with HTS in Gene Expression Omnibus (GEO, https://www.ncbi.nlm.nih.gov/gds/?term=) database were selected following the selection criteria: 1) Independent expression profiles of HTS; 2) Both HTS and normal controls were involved; 3) Total sample size in each profile was more than 6; 4) Test specimens from datasets of human (Homo sapiens) skin tissues. Finally, three datasets including GSE178411 (https://www.ncbi.nlm.nih.gov/geo/query/acc.cgi?acc=GSE178411), GSE181540 (https://www.ncbi.nlm.nih.gov/geo/query/acc.cgi?acc=GSE181540) and GSE188952 (https://www.ncbi.nlm.nih.gov/geo/query/acc.cgi?acc=GSE188952) were downloaded. GSE178411 and GSE181540 served as training datasets, while GSE188952 was used as the validation dataset in this study. Bridely, GSE178411 included data from 28 HTS samples and 24 normal controls, based on GPL24676 platform. GSE181540 included data from 3 HTS samples and 3 normal controls, based on GPL20301 platform. GSE188952 included data from 5 HTS samples and 3 normal controls, based on GPL16791 platform.

To ensure data comparability and analytical reliability, the following preprocessing steps were performed after downloading the raw CEL files in this study: First, background correction and standardization were conducted using the Robust Multi-array Average (RMA) method from the R package affy, which included background correction, quantile normalization, and log2 transformation to reduce technical noise. Second, probe annotation and gene merging were carried out by mapping probes to gene symbols based on the latest annotation files. For multiple probes corresponding to the same gene, the average expression value was taken as the representative. Probes without matching gene symbols were eliminated using the probe expression matrix and annotation file. Third, missing value handling involved removing probes with a missing value ratio exceeding 20% and imputing sporadic missing values using the k-nearest neighbors (KNN) imputation method. Fourth, if the data originated from multiple experimental batches, batch effect correction was performed using the ComBat method in the sva package (version 4.3.3). Finally, low-expression gene filtering was implemented to remove genes whose expression values fell within the lowest 25th percentile in more than 80% of the samples, thereby reducing interference from low-information features in the analysis.

### 2.2 DEGs, GSEA and immune landscape analysis

We employed the limma package in R (version 3.58.1) (15) to identify differentially expressed genes (DEGs) between HTS and normal samples across two training datasets. First, we performed significance analysis of gene expression using log2 fold change (FC) and *P*-values, selecting DEGs with Benjamini-Hochberg (BH)-adjusted (adj) *P* < 0.05 and |log_2_FC| > 1. Next, Gene Set Enrichment Analysis (GSEA) (16) was conducted with thresholds of adjusted *P* < 0.05 and NES > 1 to identify significant pathways. The ssGSEA algorithm (17) was then applied to assess infiltration levels of 28 immune cell subtypes based on training set expression profiles. Finally, we employed the ggplot2 (version 3.5.0) package in R to calculate the Pearson correlation coefficient, analyzing correlations among immune cells.

### 2.3 WGCNA analysis

The analysis commenced with performing variance analysis on the HTS sample expression matrix of to identify the top 5000 most variable genes. We further employed WGCNA (version 1.72-5) to construct a gene co-expression network. Specifically, it can transform high-dimensional transcriptome data into gene modules, revealing gene clusters with similar expression patterns and their underlying biological functions. Meanwhile, it enables direct correlation analysis between co-expression modules and phenotypes, facilitating the identification of gene groups significantly associated with immune cells. Additionally, through modular analysis, WGCNA reduces the number of statistical tests, thereby enhancing the robustness of results. Furthermore, it is well-suited for large-scale data, as it maintains high computational efficiency and interpretability even when clustering 5,000 genes.

At first, the soft threshold was employed to transform the adjacency matrix into a continuous range from 1 to 30. This was done to guarantee that the resultant network conformed to a power-law distribution, which better represents the characteristics of biological networks. In WGCNA analysis, module selection adheres to the following criteria: first, the soft threshold (β) was identified via the pickSoftThreshold function, selecting the smallest β value that enables the network to approximate scale-free topology (typically with R² ≥ 0.85); second, the minimum module size was set to 100 genes to avoid statistical instability caused by excessively small modules; finally, Pearson correlation coefficients and p-values between each module eigengene and immune cells were computed, with modules meeting |correlation| ≥ 0.75 and p < 0.05 prioritized for subsequent analysis. After that, the blockwiseModules function was used to generate the scale-free network, and module partition analysis was carried out. For each module, the module membership and gene significance were computed.

### 2.4 Investigation of immune-inflammation gene associated HTS

Inflammation-related genes were retrieved from the MSigDB database, with subsequent removal of duplicates entries. Then, the cross genes among DEGs, modules genes in WGCNA and inflammation-related genes were revealed by using Venn diagram analysis.

### 2.5 The enrichment and interaction analysis based on cross genes

GO function and KEGG pathway analyses were implemented using the clusterProfiler package in R for intersecting genes. The GO function encompasses biological process (BP), cellular component (CC), and molecular function (MF). For the current enrichment analysis, an adjusted P value less than 0.05 was set as the threshold. Subsequently, based on the STING database (version: 11.0) (18), the protein interaction information was retrieved. Protein-protein interaction (PPI) pairs among the differentially expressed high-mobility group proteins (DE-HMGs) were predicted, with a combined_score greater than 0.4. The PPI network was built using Cytoscape (version: 3.8.2) software. The maximum clique centrality (MCC), maximum neighborhood component (MNC), degree centrality (Degree), and edge percolated component (EPC) topology algorithms within the cytoHubba package of the Cytoscape software were applied. These algorithms were used to identify hub genes, taking into account the TOP 30 nodes in the PPI network.

### 2.6 Signature genes exploration and evaluation

In the feature screening stage, LASSO regression was used for dimensionality reduction of high-dimensional expression data—it achieves automatic feature selection by compressing some regression coefficients to zero through L1 regularization during fitting, which can effectively reduce redundant variables and mitigate the impact of multicollinearity. Specifically, the R package glmnet was used to perform 10-fold cross-validation under a series of regularization parameters λ, and the feature set corresponding to lambda.min (the λ with the minimum cross-validation error) was selected as input for subsequent modeling. In the classification stage, support Vector Machines (SVM) was employed to build a prediction model, which has strong generalization ability in the analysis of high-dimensional, small-sample, and noisy biological data, and is particularly suitable for problems with nonlinear decision boundaries. Therefore, the radial basis function (RBF) kernel was prioritized to capture complex nonlinear patterns, and the penalty coefficient C and kernel parameter γ were optimized through grid search combined with cross-validation. To further evaluate feature stability and model generalization performance, random forest (RF) was simultaneously used for modeling and feature importance ranking. RF stably handles high-dimensional data and calculates variable importance scores by constructing multiple decision trees and using out-of-bag (OOB) data for error estimation, the parameter optimization of which involves adjusting the number of decision trees (ntree) and the number of random features (mtry) through grid search, selecting the parameter combination with the lowest OOB error. To ensure model robustness and generalizability, all algorithms adopt 10-fold cross-validation: the data is randomly divided into 10 subsets, with one subset used as the validation set and the remaining nine as the training set in turn. Within each fold, feature selection, model training, and parameter optimization were performed in sequence to prevent data leakage, and grid search was conducted to optimize key hyperparameters for LASSO (λ), SVM (C/γ), and RF (ntree/mtry) respectively, with the cross-validation process repeated more than 100 times. Additionally, measures such as separating feature selection from model training to avoid leakage of training set information, comparing multiple algorithms (SVM and RF) to verify feature reproducibility, randomly dividing training/validation sets multiple times to ensure results do not depend on a single data split, and combining OOB error with cross-validation for dual evaluation of model performance further guarantee the robustness and reliability of the models.

The genes that were commonly identified by all three algorithms were regarded as the signature genes for HTS. Wilcoxon signed rank tests were performed to evaluate the differential expression of the signature genes between the HTS and controls, based on all training datasets and the validation dataset. Receiver operating characteristic (ROC) curves analysis was performed using the pROC package (version 1.12.1). This was done to calculate the Area Under the Curve (AUC) value for each signature gene when comparing HTS samples with normal samples. Furthermore, the signature genes were employed for constructing a nomogram through the rms package in R. A nomogram was established based on the nomoScore values of all genes by using the rms package (version 6.3-0). In addition, a calibration curve, a decision curve, and a clinical curve were generated to assess the performance of the nomogram.

### 2.7 Integration analysis on signature genes

In this study, using GeneMANIA, a PPI network was constructed. This network incorporated feature genes and 20 interacting partners to analyze colocalization and functional correlations. The DSigDB was then utilized to build a drug-target interaction network, elucidating relationships between the feature genes and drugs. GSEA was performed for signature genes, with significant pathways defined by adjusted P-value (adj *P*) less than 0.05 and a Normalized Enrichment Score (NES) greater than 1.

### 2.8 Patients and sample collection

Tissue samples were obtained from 12 HTS patients, and these patients were recruited from the Department of Burn and Wound Repair in our Hospital. They were aged between 23 and 65. All 12 patients were found to develop HTS based on wounds. Paired samples of HTS and adjacent non-lesional skin were collected from each patient. Primary fibroblasts were isolated from both tissue types. Samples were immediately stored in sterile pre-cooled physiological saline and transported to the laboratory within 1 hour for immediate processing. This research protocol was approved by the Ethics Committee of Weifang People’s Hospital. The informed consent of all participants was obtained.

### 2.9 Cell culture

Harvested tissues from 12 HTS patients were washed thrice with PBS containing dual antibiotics (100 U/mL penicillin and 100 μg/mL streptomycin) to eliminate blood and contaminants. The tissues were then minced into tiny fragments of about 1mm³ and uniformly seeded into culture flasks. Flasks containing DMEM with 10% FBS and 1% GlutaMAX were incubated at 37°C with 5% CO_2_ for cell cultivation. Once the cells migrated from the tissue pieces and reached 90% confluence, cells were passaged using 0.25% trypsin-EDTA. Cells from the 3rd to the 5th passage were employed for the subsequent experiments.

### 2.10 Quantitative real-time PCR analysis

Total RNA was isolated from both 12 HTS tissues and normal skin tissues using TRIzol reagent (Invitrogen, USA), adhering to the manufacturer’s instructions. The concentration and purity of the extracted RNA were determined with a NanoDrop spectrophotometer (Thermo Fisher Scientific, USA). The PrimeScript RT reagent kit (Takara, Japan) was utilized to synthesize cDNA. QRT-PCR was performed on a QuantStudio 5 Real-Time PCR System (Applied Biosystems, USA) with SYBR Green Master Mix (Roche, Switzerland). Target gene expression levels were quantified, with GAPDH as the endogenous control. All primer details are provided in **Supplementary Table S1**. The relative expression levels of the diagnostic genes were computed using the 2^-ΔΔCT^ method (19).

### 2.11 Western blot analysis

Protein was extracted using RIPA lysis buffer (Beyotime, China) supplemented with protease inhibitors. Protein concentrations were measured using the BCA Protein Assay Kit (Thermo Fisher Scientific, USA). Equal quantities of protein (30 µg) were subjected to separation via SDS-PAGE and then transferred onto PVDF membranes (Millipore, USA). The membranes were blocked with 5% non-fat milk and then incubated at 4°C overnight with primary antibodies. These primary antibodies were against *COL1A1* (1:1000, Abcam, USA), *A2M* (1:1000, Abcam, USA), *TIMP1* (1:1000, Abcam, USA), *COL1A2* (1:1000, Abcam, USA), and GAPDH (1:5000, Abcam, USA). After washing, the membranes were incubated for 1 hour at room temperature with HRP-conjugated secondary antibodies (1:5000, Abcam, USA). Protein bands were visualized using an ECL detection system (Bio-Rad, USA), and the intensities of the bands were quantified using ImageJ software.

### 2.12 The siRNA transfection and efficiency evaluation

To investigate the biological function of *COL1A1*, siRNA targeting *COL1A1* (si-COL1A1) and a negative control siRNA (si-NC) were synthesized by GenePharma (China). All 12 HTS tissue-derived fibroblasts were transfected with si-COL1A1 or si-NC using Lipofectamine 3000 (Invitrogen, USA) following the manufacturer’s guidelines. Forty-eight hours post-transfection, total RNA was extracted. *COL1A1* knockdown efficiency was then verified through qRT-PCR.

### 2.13 CCK-8 assay detection of proliferation activity

Cell proliferation was evaluated using CCK-8 (Dojindo, Japan) assay. Fibroblasts transfected with si-COL1A1 or si-NC were plated into 96-well plates at a seeding density of 5×10^3^cells per well. At 0, 24, 48, and 72 hours after seeding, 10 µL of CCK-8 solution was added to each well. After incubation at 37°C for 2 hours. The absorbance at 450 nm was measured using a microplate reader (BioTek, USA). All experiment were performed in triplicate.

### 2.14 Flow cytometry detection of fibroblast apoptosis

Apoptosis was evaluated using the Annexin V-FITC/PI Apoptosis Detection Kit (BD Biosciences, USA). Fibroblasts transfected with si-COL1A1 or si-NC were collected 48 hours after the transfection process. These cells were washed with PBS and then resuspended in binding buffer. Subsequently, the cells were stained with Annexin V-FITC and PI (propidium iodide) for 15 minutes at room temperature in darkness. The apoptotic cells were analyzed using a BD FACSCalibur flow cytometer (BD Biosciences, USA). Data were analyzed using FlowJo software (Tree Star, USA).

### 2.15 Statistical analysis

Statistical analyses were carried out using GraphPad Prism 9.0 software. An unpaired t-test and Wilcoxon rank-sum test were applied to assess the differences between the two groups. Pearson’s correlation analysis was used to identify correlations between variables. All statistical tests were two-sided, and a P-value of 0.05 was considered to indicate statistical significance. The study design flowchart is provided in **Supplementary Figure S1**.

## 3 Results

### 3.1 DEGs, GSEA and immune infiltration analysis in HTS groups vs. normal groups

**Figure 1:**
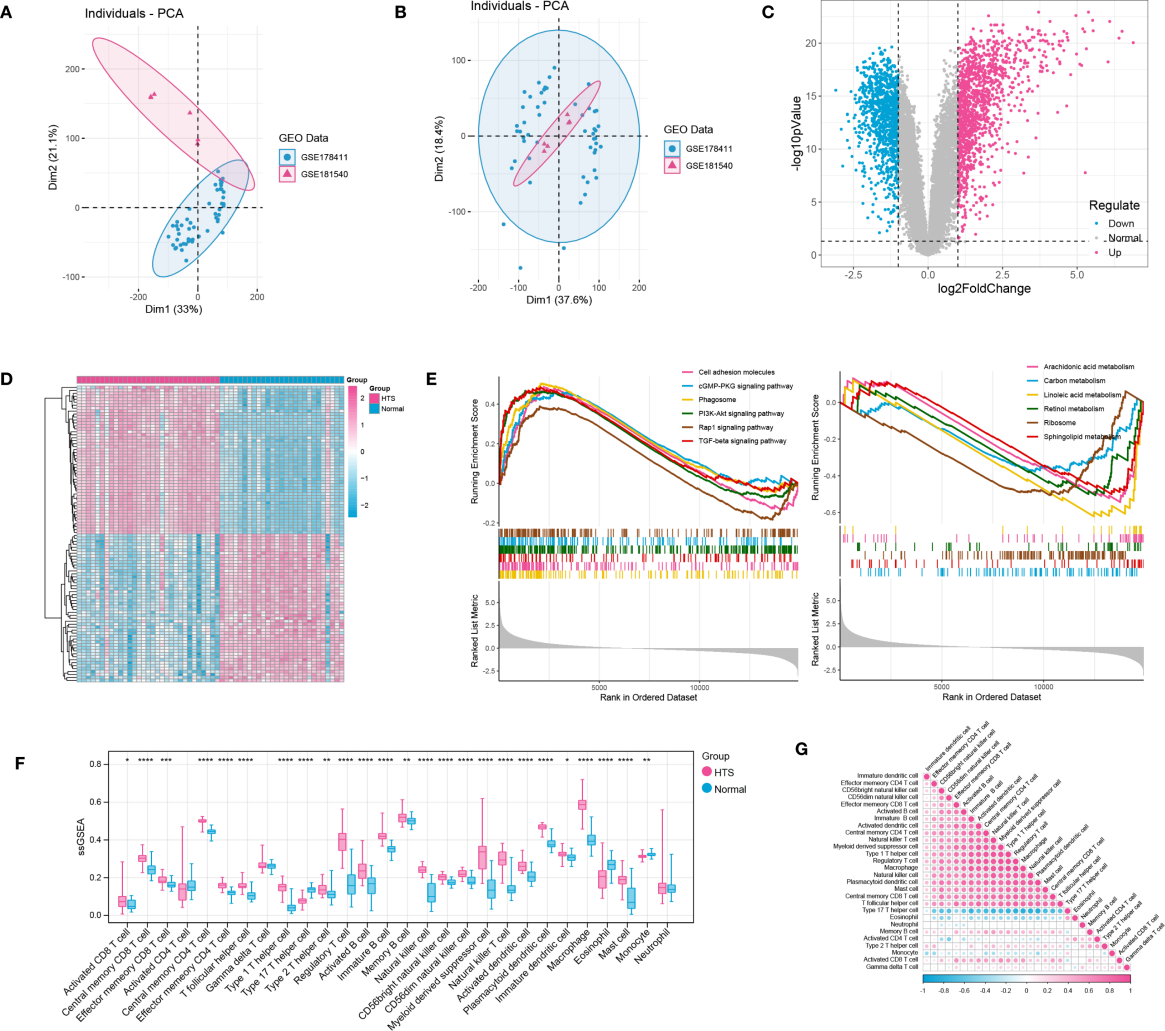
Integration investigation for hypertrophic scar (HTS) samples vs. normal samples based on training datasets. **(A, B)** PCA distribution of expressed data before and after removing batch effects in SVA. **(C)** the volcano plot showed the differentially expressed genes (DEGs) between HTS samples and normal samples. **(D)** the heatmap showed all samples could be separated by different groups (samples). **(E)** the TOP 6 significant up-regulated and down-regulated pathways between HTS group and normal group revealed by GSEA analysis. **(F)** immune infiltration analysis revealed the outstanding immune cells between HTS group and normal group. **(G)** the correlation analysis for immune cells by using heatmap analysis. *P<0.05, **P<0.01, ***P<0.001, ****P<0.0001.

After data preprocessing, a total of 31 HTS samples and 27 normal samples were enrolled based on two training datasets (**Figures 1A, B**). Then, differential expression analysis identified a total of 2244 DEGs including 1249 up-regulated genes and 995 down-regulated genes between HTS and normal samples (**Figure 1C**). The outcome of the heatmap analysis demonstrated that all the samples could be distinctly differentiated according to the various groups (**Figure 1D**). Moreover, the top 6 significant up- and down-regulated pathways between HTS and normal groups were revealed by GSEA analysis (**Figure 1E**). The result showed that significantly dysregulated KEGG pathways were predominantly associated with immune response, inflammatory processes, and metabolic regulation, suggesting their potential roles in the pathogenesis of HTS. Finally, based on ssGSEA algorithm, comparative analysis revealed 25 differentially infiltrated immune cells (DICs) between HTS and normal groups with *P* < 0.05 (**Figure 1F**). For example, activated B cells showed significantly increased infiltration in HTS compared to normal controls (*P* < 0.0001). Furthermore, the correlation analysis revealed predominantly significant positive associations among the majority of immune cell types, suggesting a coordinated immune response in the tissue microenvironment (**Figure 1G**).

### 3.2 WGCNA analysis and DE-HMGs investigation

**Figure 2:**
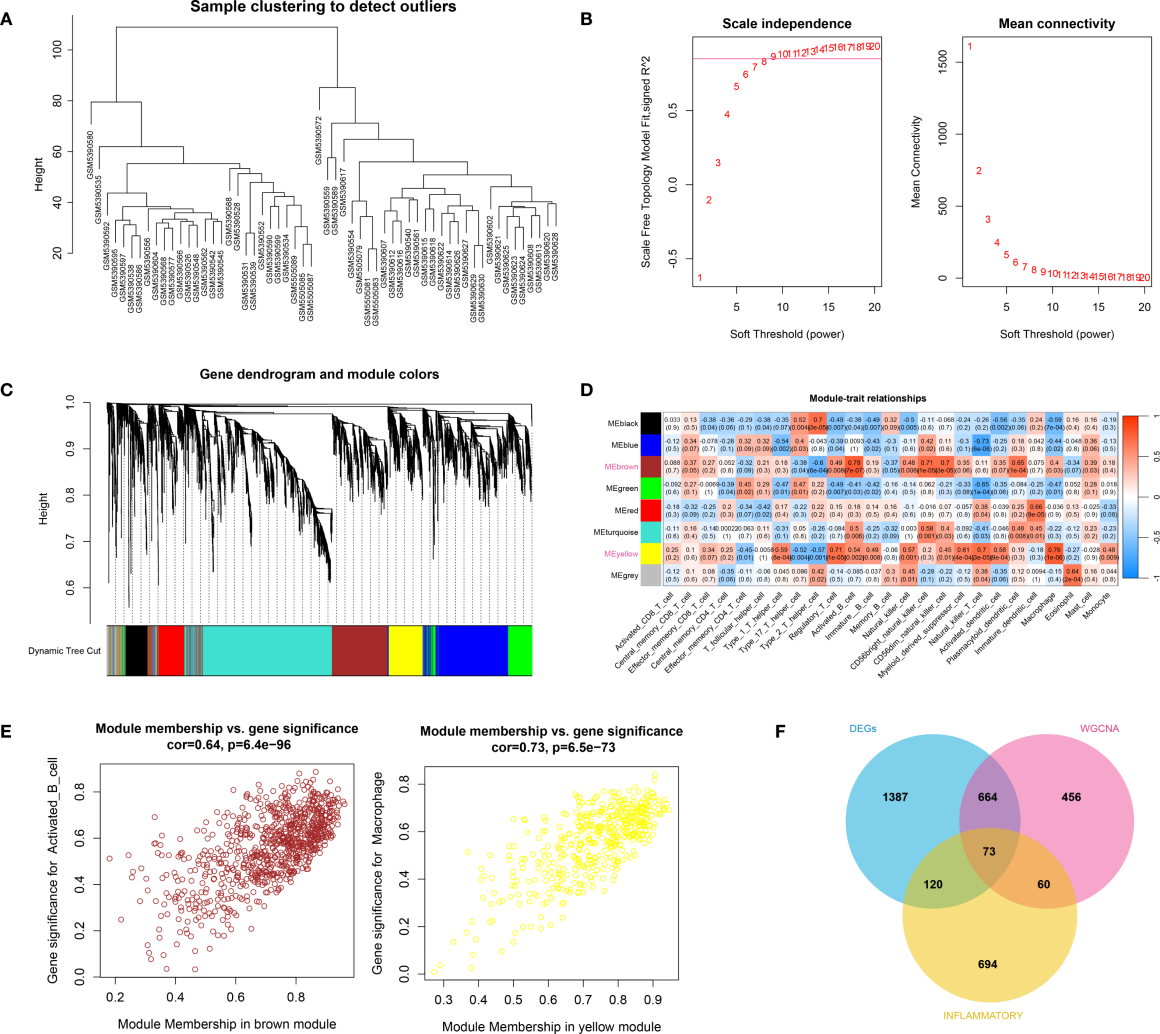
The result of WCGNA analysis. **(A)** the hierarchical clustering analysis based on the TOP5000 genes. **(B)** the scale free soft-threshold distribution. **(C)** clustering analysis for models: dynamic tree cut and merged dynamic represented the module before and after the merge module; the different colors in the figure represented different modules. **(D)** the heatmap for correlation between modules and traits. **(E)** the gene significant analysis for genes in brown module and yellow module. **(F)** the VENN plot analysis revealed 73 common genes (cross genes) for HTS among DEGs, inflammation-related genes and module genes.

The hierarchical clustering results based on the top 5000 genes demonstrated good data quality and reliability, providing a solid foundation for subsequent analysis (**Figure 2A**). Then, WGCNA analysis was conducted using a soft-threshold of 8 and a fitting degree of 0.85 (**Figure 2B**). Consequently, two modules were identified based on the combination results of dynamic tree cutting (**Figure 2C**). The correlation between the modules and different groups was visualized with a heatmap (**Figure 2D**). The analysis revealed that the black module (r = 0.51, *P* < 0.001) and brown module (r = 0.5, *P* < 0.001) had the strongest positive correlations with HTS, identifying them as key modules in this study. Gene significant analysis demonstrated a strong correlation among module genes and HTS (**Figure 2E**). Thus, these 1253 module genes were enrolled for the following analysis. Furthermore, the VENN plot analysis identified a total of 73 common genes among DEGs, modules genes and inflammation-related genes (**Figure 2F**), which were used as cross genes for following analysis.

### 3.3 Cross gene and associated analysis

**Figure 3:**
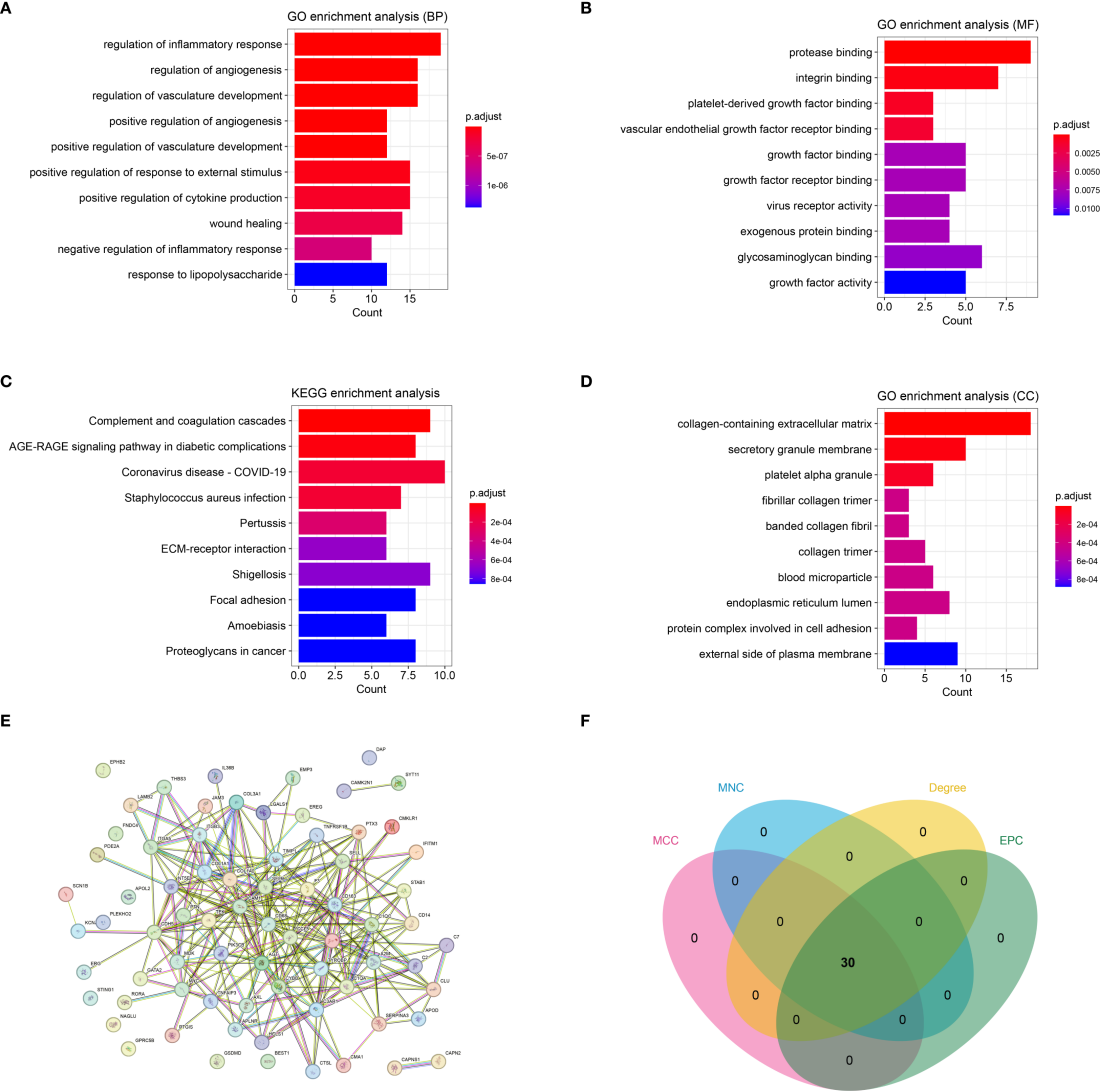
The immune-inflammation associated HTS genes (cross genes) and associated analysis. **(A)** the significant GO-BP functions assembled by cross genes. **(B)** the significant GO-MF functions assembled by cross genes. **(C)** the significant GO-CC functions assembled by cross genes. **(D)** the significant KEGG pathways enriched by cross genes. **(E)** the protein-protein interaction network constructed by cross genes. **(F)** the VENN plot revealed 30 key genes related with HTS based on four topology analysis.

By intersecting DEGs, WGCNA module genes, and inflammatory genes from databases, a total of 73 immune-inflammatory-related genes differentially expressed in HTS were identified (**Supplementary Figure S2**). Subsequently, the GO and KEGG enrichment analyses revealed that these genes were predominantly grouped into functions such as regulation of the inflammatory response (BP, GO:0050727) (**Figure 3A**), protease binding (MF, GO:0002020) (**Figure 3B**) and collagen-containing extracellular matrix (CC, GO:0062023) (**Figure 3C**). Meanwhile, these genes were mainly enriched in pathways including complement and coagulation cascades (hsa04610) (**Figure 3D**). Furthermore, the PPI analysis revealed a network established by 63 nodes (genes) and 254 interactions (**Figure 3E**). In addition, the topology analysis based on MCC, MNC, EPC and Degree algorithms explored 30 key genes from top 30 nodes in the PPI network (**Figure 3F**). Notably, the intersection of the top 30 genes identified by each algorithm remained 30, indicating high consistency in their prioritization.

### 3.4 Signature genes investigation and evaluation

**Figure 4:**
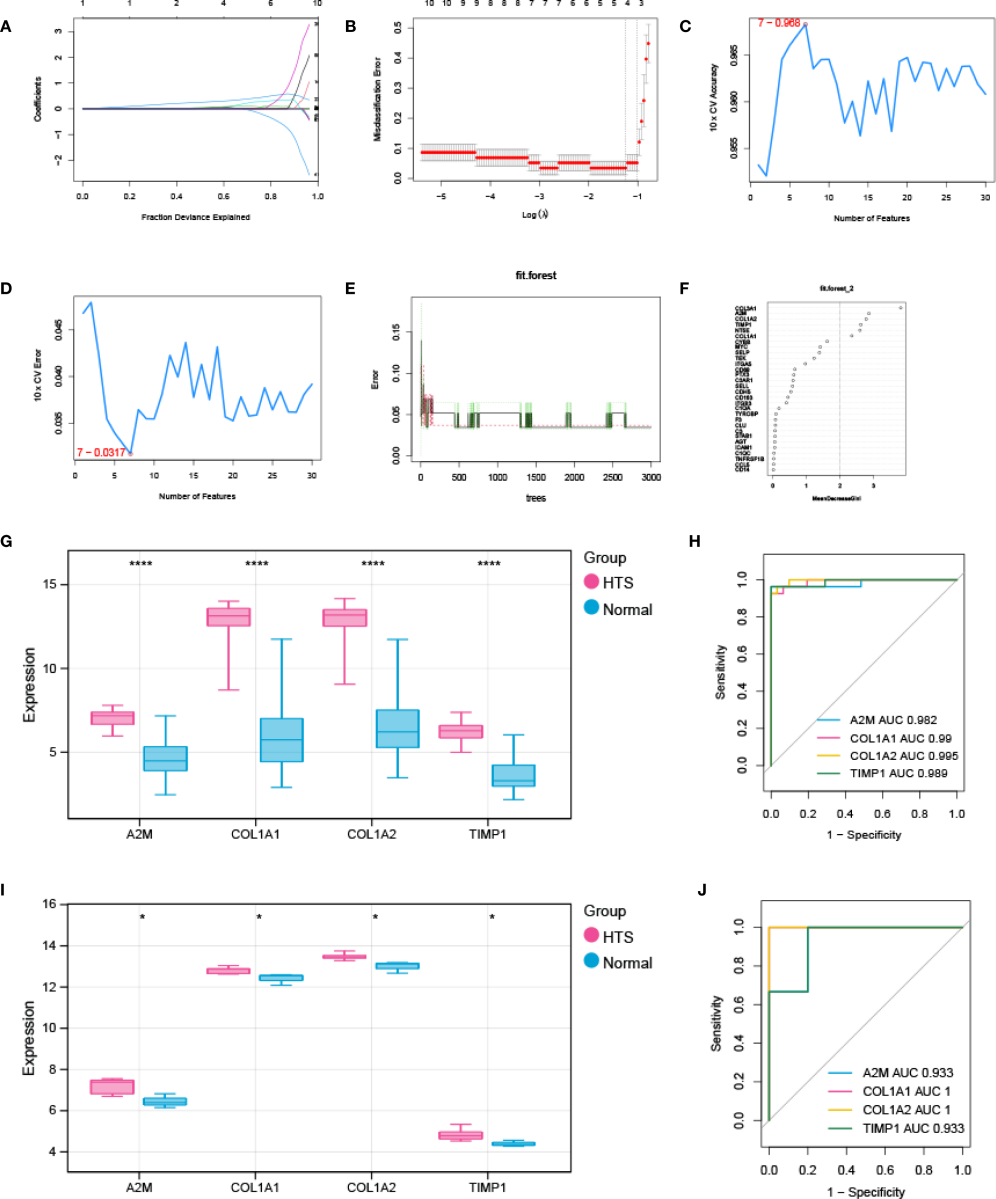
Signature genes investigation and evaluation. **(A, B)** LASSO Cox analysis revealed nine optimal genes: the Y-axis in the A represented the coefficient of the variable, while the X-axis represented the value of log (lambda); the two dotted lines in B represented two special lambda values: lambda.min on the left and lambda.1se on the right; the lambda between these two values was considered appropriate. **(C)** the accuracy for SVM-RFE analysis. **(D)** the error rate for SVM-RFE analysis. **(E)** relationship between the number of trees and the error rate in a RF model. **(F)** the Top 6 genes selected by using RF algorithm. **(G, H)** the validation analysis for signature genes based on training datasets: box plot in G showed the expression of signature genes between EM group and control group, while the ORC curve in H represented the AUC value for all signature genes. **(I, J)** the validation analysis for signature genes based on validation dataset. *P < 0.05; ****P < 0.0001.

Three advanced machine learning algorithms were employed to analyze the 30 key genes. The LASSO regression analysis successfully singled out five significant genes (**Figures 4A, B**). At the same time, the SVM-RFE method identified seven genes (**Figures 4C, D**). Moreover, the Random Forest algorithm, utilizing a MeanDecreaseGini threshold greater than 2, uncovered the top six genes (**Figures 4E, F**). The intersection of genes selected by these various methods resulted in a final set of four signature genes, including *A2M*, *COL1A1*, *COL1A2* and *TIMP1*, for investigation of HTS (**Supplementary Figure S3**). The evaluation analysis was performed on four signature genes. The results showed that all signature genes were significantly up-regulated in the HTS group when compared to the normal group in the training dataset (**Figure 4G**), and ROC analysis showed that AUC values for each gene were larger than 0.982 in both training dataset (**Figure 4H**). In addition, the analysis based on the validation gene set further confirmed the results obtained from the training set (**Figures 4I, J**).

### 3.5 Nomogram investigation based on signature genes

**Figure 5:**
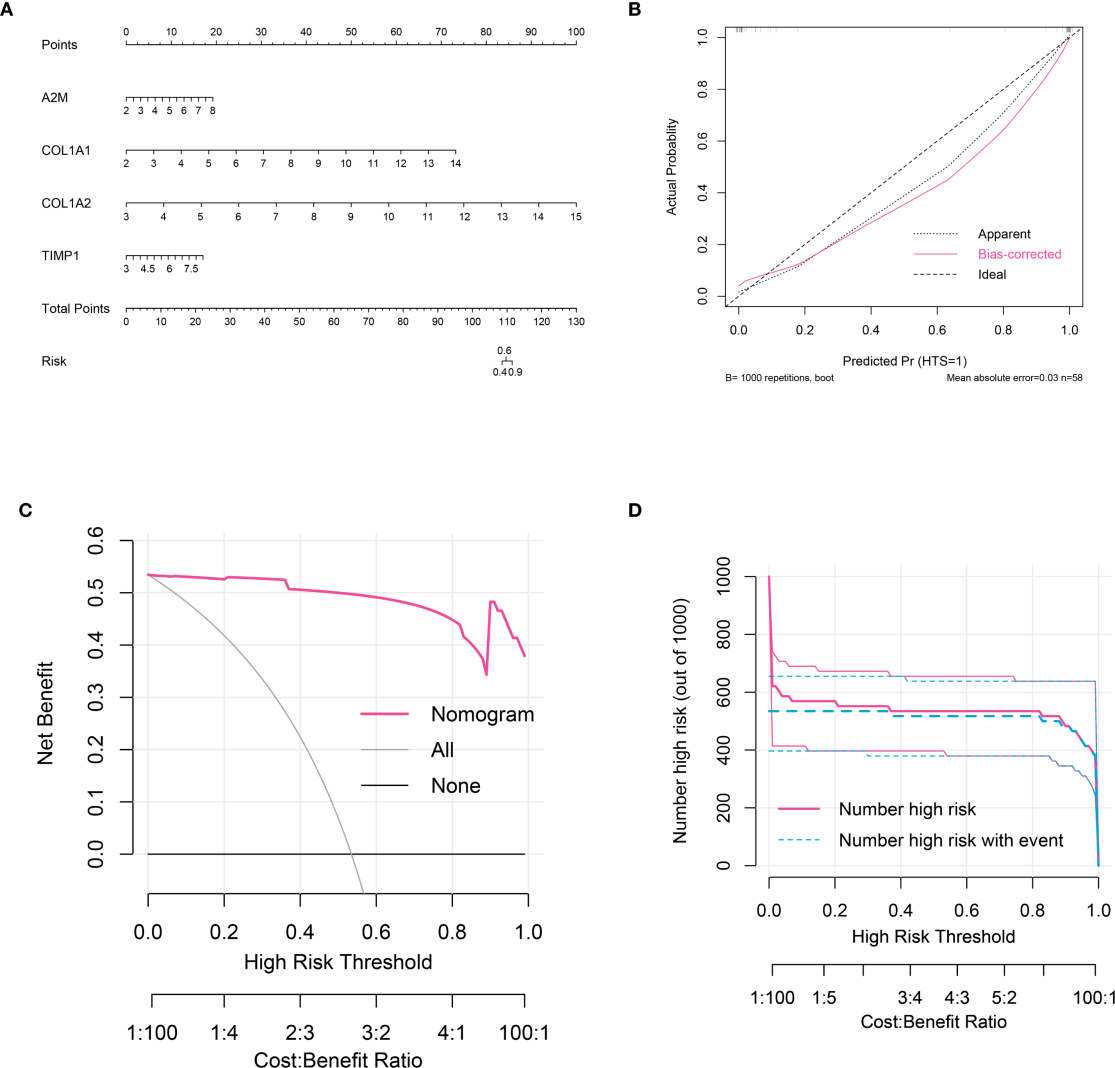
Nomogram analysis for signature genes. **(A)** nomogram model constructed by four signature genes predicting the risk of HTS: the nomogram was used by summing all points identified on the scale for each variable; the total points projected on the bottom scales indicate the risk of HTS. **(B)** calibration curve analysis to validate the predicable of nomogram. **(C)** the decision curve analysis used to evaluate optimal threshold for current nomogram. **(D)** the clinical curve for evaluating the predictive power of nomograph model.

A nomogram was established using grouping information and expression of six signature genes (**Figure 5A**). Each variable was scored on the point scale axis. The analyses of calibration curve (**Figure 5B**), decision curve (**Figure 5C**) and clinical curve (**Figure 5D**) showed the margin of error between actual HTS risk and predicted risk was minimal, suggesting high prediction accuracy for HTS within the nomogram model.

### 3.6 Integration analysis for signature genes

**Figure 6:**
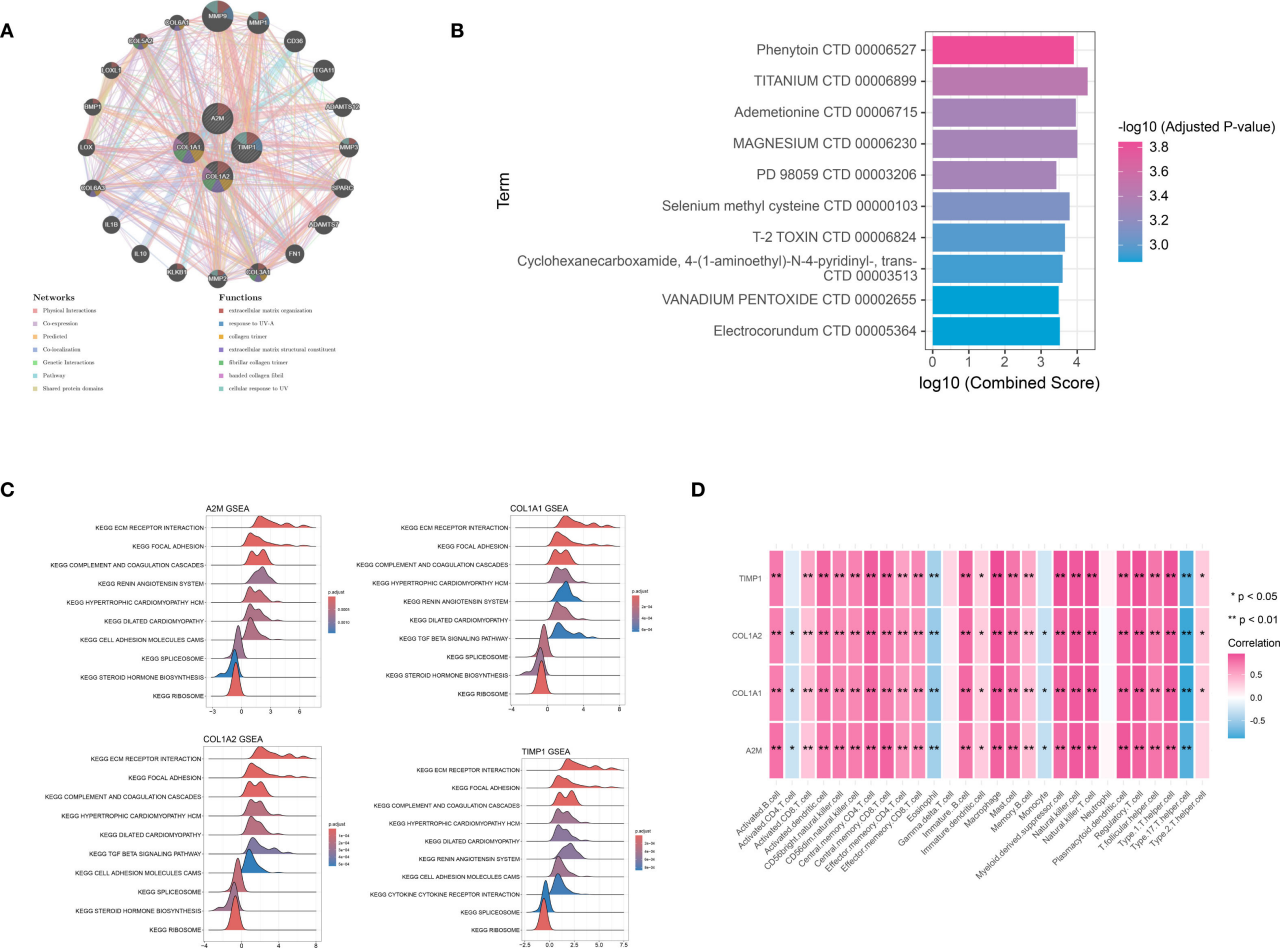
Integration analysis for signature genes. **(A)** the PPI interaction network analysis for signature genes: different circle represented different gene, while different color represented functions associated with signature gene. **(B)** the TOP 10 target drugs predicted for signature genes: the redder the color, the more significant the P value. **(C)** the Gene Set Enrichment Analysis (GSEA) analysis based on signature genes. **(D)** immune correlation analysis for signature genes. *P < 0.05; **P < 0.01.

The GeneMANIA database was utilized to analyze the functional interactions of the signature genes, resulting in the construction of a gene-gene interaction network. The network displayed four signature gene nodes encircled by 20 nodes representing genes with significant associations, suggesting potential functional linkages and regulatory relationships (**Figure 6A**). Based on the DSigDB database, we identified four signature genes and their interacting drugs. Notably, compounds such as Phenytoin (CTD 00006527) and TITANIUM (CTD 00006899) showed strong interactions with the signature genes, suggesting potential therapeutic relevance (**Figure 6B**). Moreover, the GSEA analysis showed that all signature genes were enriched in pathways such as the extracellular matrix (ECM) receptor interaction and ribosome (**Figure 6C**). Furthermore, the immune correlation analysis we performed showed that signature genes were significantly correlated with immune cells like Natural killer T cells (all *P* < 0.05), which collectively contribute to the immune microenvironment (**Figure 6D**).

### 3.7 Signature genes were differentially expressed between HTS tissue and normal skin tissue

**Figure 7:**
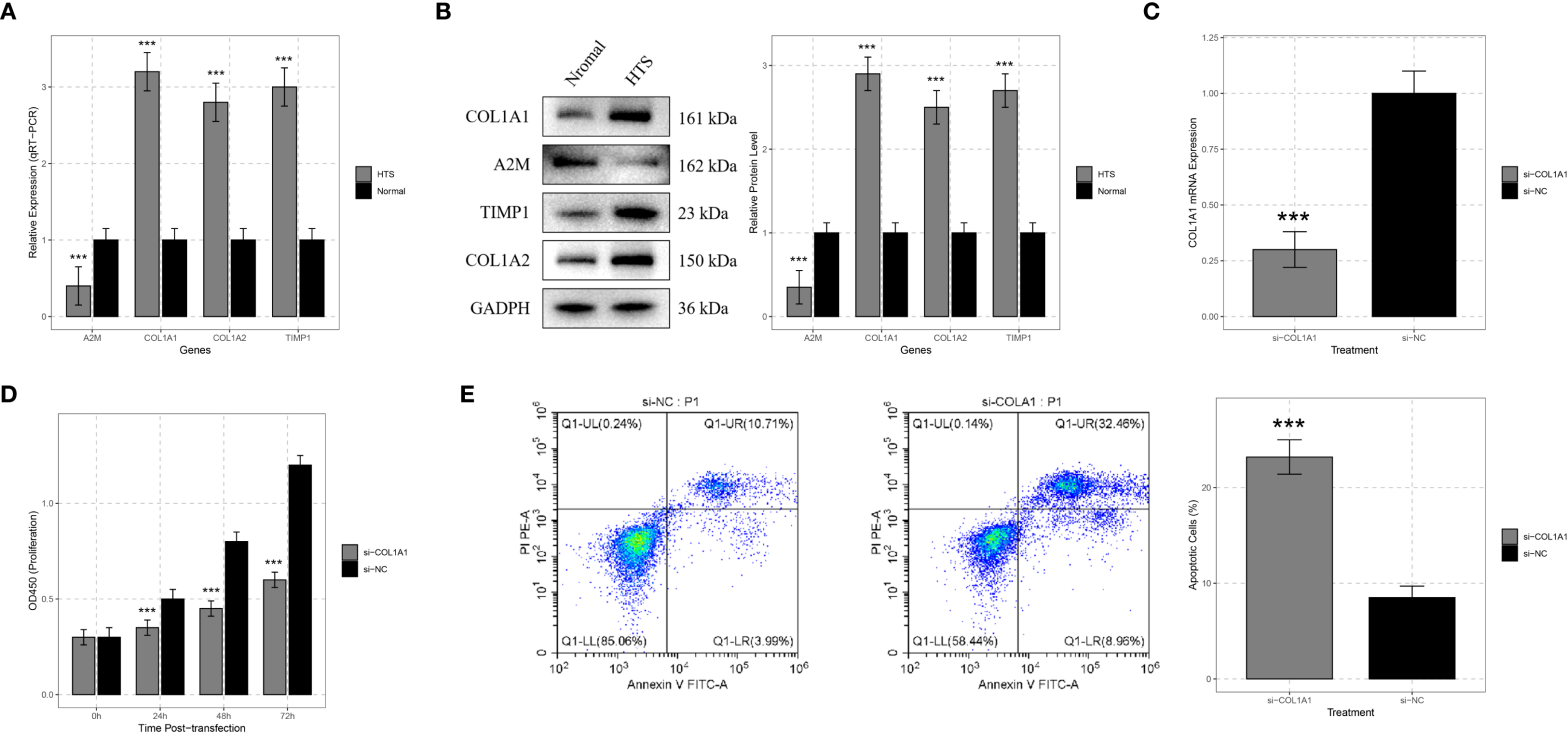
Validation analysis for signature genes. **(A)** qRT-PCR analysis showed significant differences in the expression levels of signature genes including *COL1A1*, *A2M*, *TIMP1*, and *COL1A2* between hypertrophic scar (HTS) tissue and normal skin tissue. **(B)** Western blot analysis was carried out on samples from HTS tissue and normal skin tissue to confirm the expression differences of *COL1A1*, *A2M*, *TIMP1*, and *COL1A2* at the protein level. **(C)** to verify the efficiency of siRNA - mediated knockdown, HTS fibroblasts were transfected with si-COL1A1, and the change in *COL1A1* mRNA expression was compared with the si-negative control (si-NC) group. **(D)** CCK-8 assays were conducted on HTS fibroblasts transfected with si-COL1A1 to determine the impact of *COL1A1* knockdown on cell proliferation at 24, 48, and 72 hours post-transfection. **(E)** flow cytometry analysis was applied to HTS fibroblasts transfected with si-*COL1A1* to evaluate the effect of *COL1A1* knockdown on apoptosis, with comparison to the si-NC group. ***P < 0.001.

The expression levels of *A2M*, *COL1A1*, *COL1A2*, and *TIMP1* in 12 HTS tissues and normal skin tissues were analyzed using qRT-PCR and Western blot. The results of qRT-PCR demonstrated that compared to normal skin tissues, the expression of *A2M*, *COL1A1*, *COL1A2*, and *TIMP1* was significantly upregulated in HTS tissues (all *P* < 0.001) (**Figure 7A**). Western blot analysis revealed that the protein levels of *A2M*, *COL1A1*, *COL1A2*, and *TIMP1* were significantly elevated in HTS tissues compared to normal skin tissues (**Figure 7B**).

### 3.8 COL1A1 promoted fibroblast proliferation in HTS

A previous study demonstrated that *COL1A1* was significantly dysregulated in HTS tissues (20). Existing studies have revealed that *COL1A1* plays a crucial role in the process of tissue fibrosis by directly regulating collagen fibril formation (21). This is highly consistent with the major pathological features of HTS. Therefore, COL1A1 was selected as the target gene for following functional validation.

To deeply explore the function of the *COL1A1* gene in HTS, we transfected si-COL1A1 into HTS fibroblasts. Compared with the group transfected with negative control si-NC, *COL1A1* mRNA expression was significantly reduced after transfection with si-COL1A1 (*P* < 0.001), which confirmed the efficiency of the siRNA-mediated gene knockdown (**Figure 7C**). The CCK-8 assay was utilized to evaluate the effect of *COL1A1* gene knockdown on the proliferation ability of HTS fibroblasts. The results indicated that the knockdown of *COL1A1* significantly inhibited the proliferation of HTS fibroblasts (*P* < 0.05) (**Figure 7D**). The proliferation rate of cells transfected with si-COL1A1 was markedly lower than that of si-NC-transfected cells, suggesting that *COL1A1* plays a role in promoting the proliferation of fibroblasts in HTS.

### 3.9 COL1A1 suppressed fibroblast apoptosis in HTS

In addition, flow cytometry analysis was performed to detect the apoptosis. The results showed that compared with the si-NC group, the knockdown of *COL1A1* significantly increased the proportion of apoptotic cells (*P* < 0.001) (**Figure 7E**). These results implied that *COL1A1* exerted an anti-apoptotic effect in HTS fibroblasts.

## 4 Discussion

HTS is a pathological state marked by an overactive state of fibroblasts, abnormal accumulation of extracellular matrix, and the continuous presence of an inflammatory response. Notably, immune and inflammatory responses play pivotal roles in the formation and progression of HTS (22). Based on differential expression analysis and GSEA, this study revealed significantly up-regulated immune- and inflammation-related pathways in HTS, including the complement and coagulation cascades, as well as ECM-receptor interactions. Indeed, the activation of the complement system has been demonstrated to promote fibroblast proliferation and collagen deposition, while dysregulation of ECM-receptor interactions directly contributes to tissue fibrosis in HTS. A previous study demonstrated that *COL1A1* and *COL1A2* directly facilitate collagen fibril formation, leading to ECM deposition (23). Meanwhile, dysregulation of *TIMP1* may exacerbate fibrosis by reducing the inhibition of matrix metalloproteinases (MMPs), thereby disrupting the crucial MMP-TIMP balance that is essential for ECM turnover (24). In addition, the immunoregulatory function of *A2M* may be impaired in HTS, potentially leading to uncontrolled inflammation and fibroblast activation (25). These processes are further supported by the GSEA results from the present study, which highlight significant enrichment of ECM-receptor interactions and complement cascades and pathways that are intricately interconnected with both fibrotic processes and immune responses (26). Notably, the immune correlation analysis in the current study revealed a strong association between the signature genes and infiltrating immune cells, such as natural killer T cells and activated B cells. This might indicate the existence of a feedforward loop, in which cytokines derived from immune cells induce fibroblast activation and collagen production, while the ECM components regulate the recruitment and polarization of immune cells. These interactions emphasize the importance of immune and fibrotic components in targeted HTS therapy, providing a new perspective for understanding the immune microenvironment of HTS.

*COL1A1* encodes the α1 chain of type I collagen, a major component of the ECM critical that is critical for maintaining skin structure and facilitating wound healing (27). *COL1A1* drives fibroblast activation and excessive collagen deposition, contributing to scar hypertrophy and ECM remodeling (7). Previous evidence showed that the roles of multiple molecules in the proliferation of HTS fibroblasts were mediated by Col1A1 (20, 28). Additionally, it has been demonstrated as a valuable indicator of immune cell infiltration and the expression of immune-related genes (29). A2M is a multifunctional protease inhibitor and acute-phase reactant involved in immune regulation, cytokine sequestration, and inflammation resolution (30). Reduced A2M expression in HTS suggests impaired immunosuppressive function, potentially exacerbating inflammatory responses and fibroblast activation (25). *TIMP1*, as a metalloproteinase inhibitor that regulates ECM degradation by inhibiting MMPs (31), has recently emerged as a potential immune functional biomarker by interacting with multiple co-receptors and cell-surface receptors, including CD74, LRP1, and CD63 (32, 33). In the formation of HTS, the levels of TIMP-1 served as an important mediator for endostar (34). *COL1A2* encodes the α2 chain of type I collagen, forming heterotrimeric fibrils essential for skin tensile strength (35). Loss of Smad-interacting protein 1 leads to the upregulation of COL1A2, resulting in excessive accumulation of the ECM and promoting scar formation (36). *COL1A2* acts in concert with COL1A1 to boost collagen biosynthesis and drive the formation of fibrotic scars (37). In this study, the four signature genes that were closely related to HTS were screened out through WGCNA and machine learning algorithms. In addition, qRT-PCR analysis showed up-regulation of *COL1A1, COL1A2 and TIMP1*, and down-regulation of *A2M* in HTS tissues compared to normal skin tissues. Western blot analysis further verified these expression patterns at the protein level. These findings not only reveal the key molecular mechanisms of HTS but also provide potential drug targets for clinical treatment.

In addition, we constructed a nomogram model based on core genes, which can effectively predict the risk of HTS occurrence. As an intuitive prediction tool, the nomogram has been widely applied in the clinical diagnosis of various diseases (38). However, the clinical application of nomogram on HTS is rare. Our model, by integrating the expression levels of multiple genes, could accurately predict the risk of HTS occurrence. The predictive performance of the model has been validated through a calibration curve and decision curve analyses. The construction of this model provides a new tool for the early diagnosis of HTS and offers potential reference for personalized treatment.

Notably, there are some limitations should be noted. First, the sample size for validation is relatively small. Although qRT-PCR, Western blot, and cell experiments confirmed the differential expression of signature genes and the role of COL1A1, the limited number of HTS patient tissue samples and fibroblasts used may restrict the generalizability of the findings, failing to fully reflect the heterogeneity across different populations or clinical subtypes of HTS. Second, the study relies heavily on publicly available microarray data from the GEO database. Such data may be affected by batch effects, variations in preprocessing protocols, and incomplete clinical metadata, which could introduce biases and impact the robustness of the identified signature genes and associated mechanisms. Third, the nomogram’s generalizability across ethnicities and scar subtypes remains unknown. These limitations highlight the need for future studies with larger, multi-center validation cohorts and integrated in-house sequencing data to further verify the conclusions.

## 5 Conclusions

In conclusion, our study identified four immune-related inflammatory biomarkers for HTS, and successfully constructed a nomogram for clinical diagnosis. These findings offered new insights into HTS pathogenesis and potential therapeutic targets.

## Data Availability

The raw data supporting the conclusions of this article will be made available by the authors, without undue reservation.
