# Supplementary material for: Immune-related inflammatory gene in hypertrophic scar: prognostic and molecular mechanisms via integrated machine learning-WGCNA analysis
Source: Front Immunol. 2025 Oct 28;16:1645721. doi: 10.3389/fimmu.2025.1645721 (PMC12602242; doi:10.3389/fimmu.2025.1645721)
Supplement: Supplementary file 2 [file DataSheet2.docx]

**Supplementary Table 1 The detail information for all primers used**

**in current study**

| **Gene** | **Primer sequence**  **(5’ - 3’)** | **Product size**  **(bp)** | **Annealing temperature**  **(℃)** | **Reference sequence (NCBI)** |
| --- | --- | --- | --- | --- |
| **COL1A1** | F: GAGGGCCAAGACGAAGACAT | 140 | 60 | NM_000088.4 |
|  | R: CAGATCACGTCATCGCACAAC |  |  |  |
| **A2M** | F: AGCAGGAAGACATGAAGGGC | 110 | 60 | NM_000014.6 |
|  | R: AATCACGTCCCCGGTAGGTA |  |  |  |
| **TIMP1** | F: GCGGATACTTCCACAGGTCC | 124 | 60 | NM_003254.4 |
|  | R: GCTAAGCTCAGGCTGTTCCA |  |  |  |
| **COL1A2** | F: TGAACTTGTTGCTGAGGGCA | 138 | 60 | NM_000089.4 |
|  | R: ATATCAAGGAAGGGCAGGCG |  |  |  |
| **GAPDH** | F: GGAGCGAGATCCCTCCAAAAT | 197 | 60 | NM_002046.7 |
|  | R: GGCTGTTGTCATACTTCTCATGG |  |  |  |

**
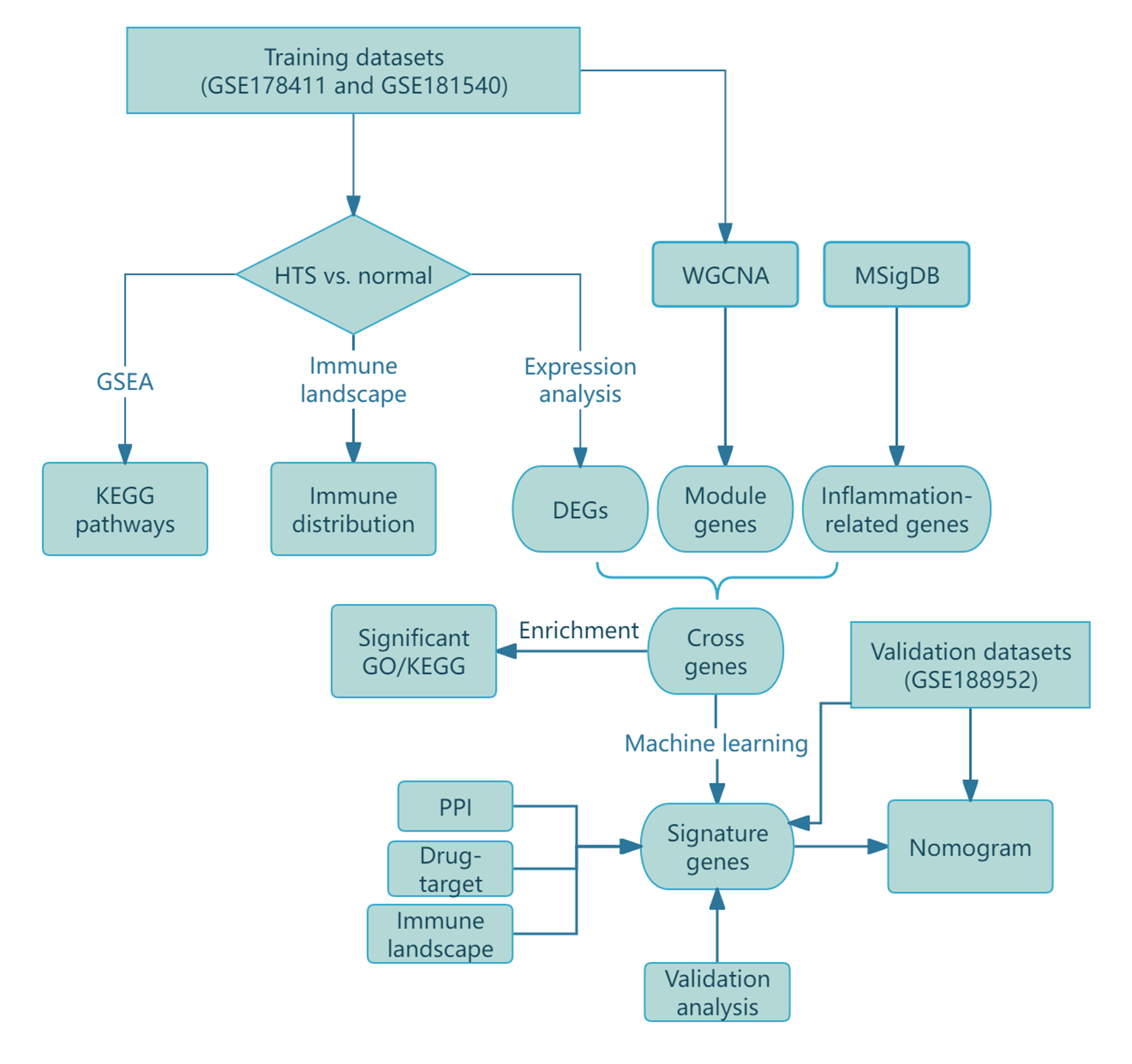
**

**Supplementary Figure 1 The flowchart for current study.**

**
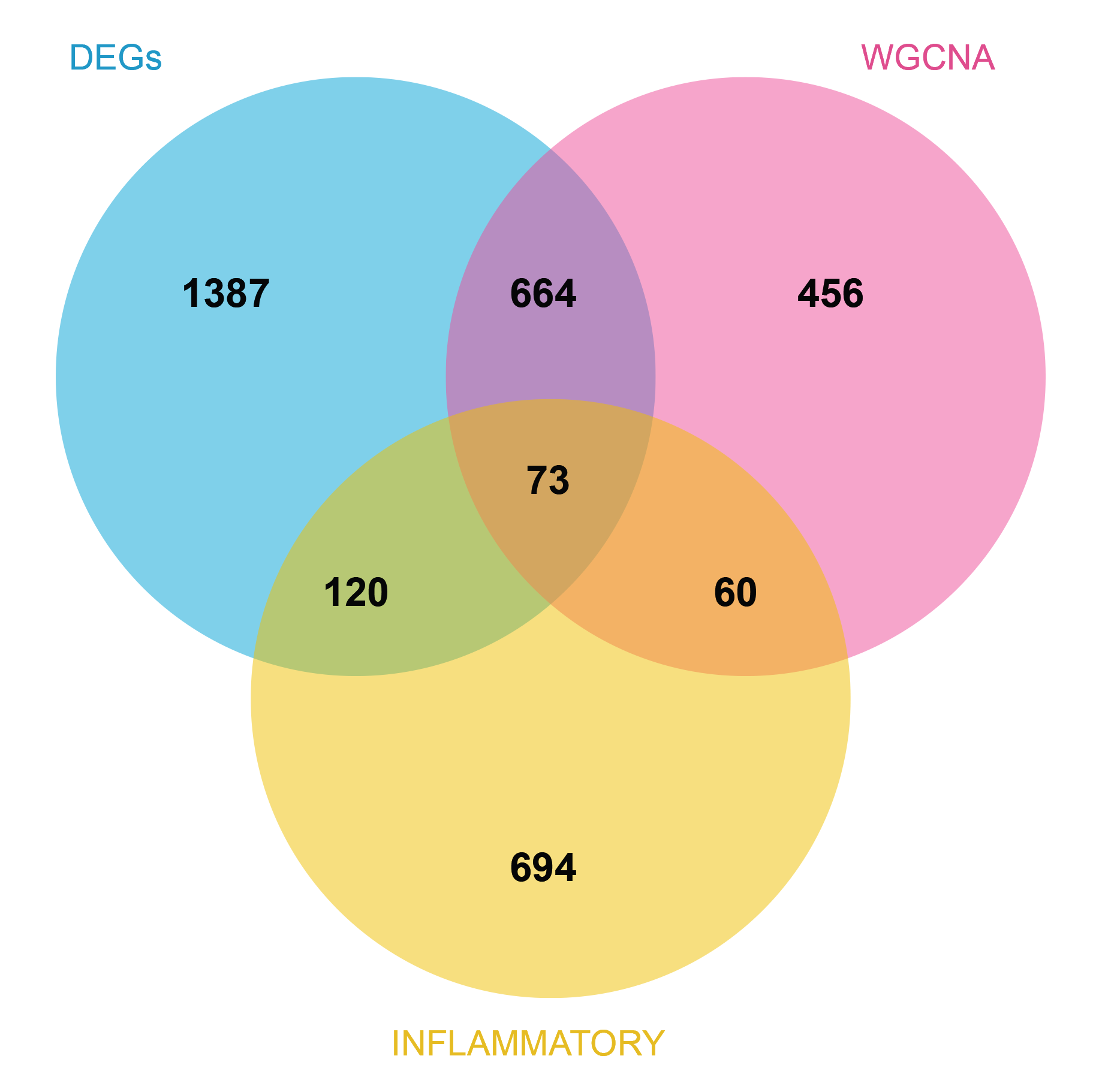
**

**Supplementary Figure 2 The VENN plot analysis revealed the cross genes in current study.**

**
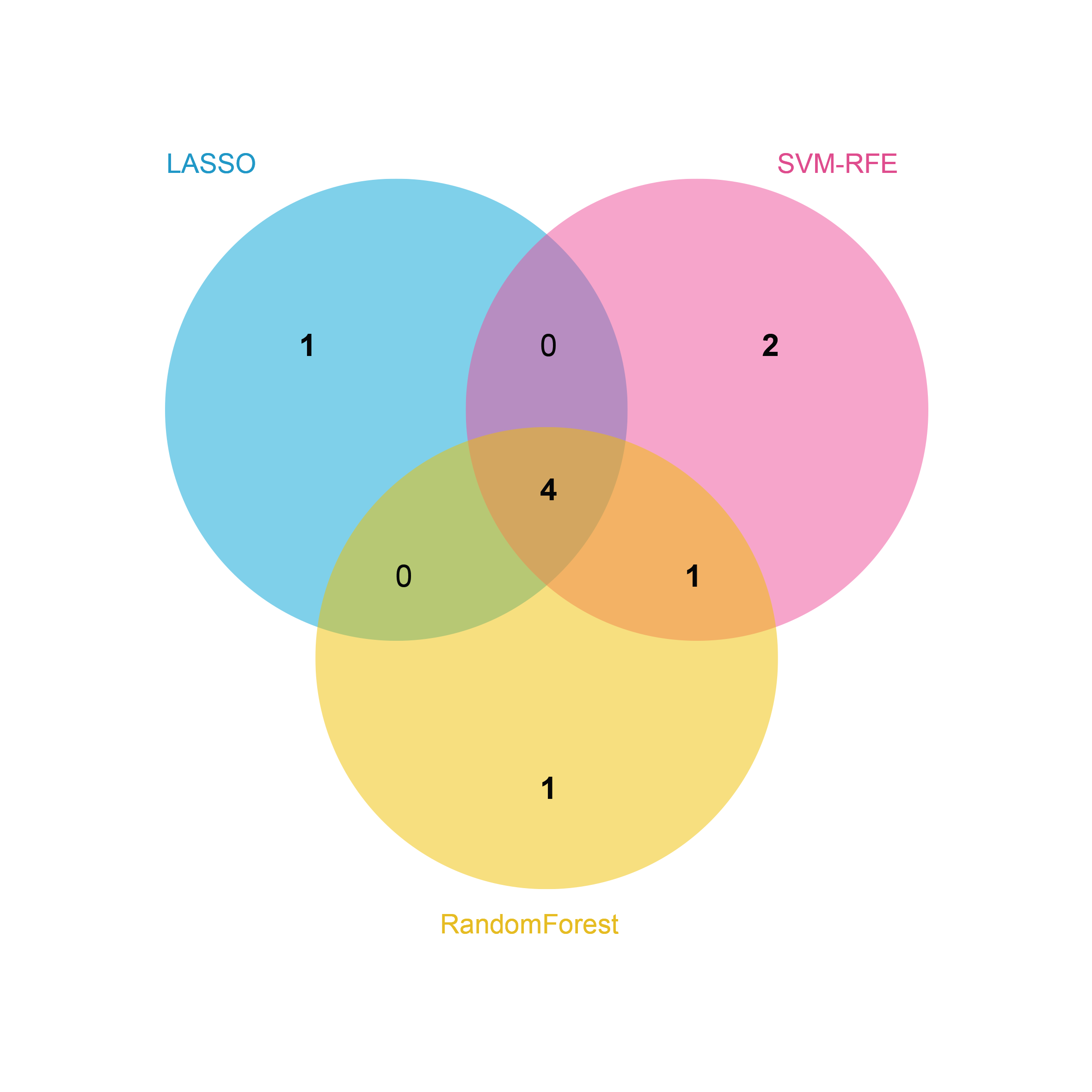
**

**Supplementary Figure 3 The VENN plot analysis showed the intersection genes selected by machine learning**
